# Hormones in malaria infection: influence on disease severity, host physiology, and therapeutic opportunities

**DOI:** 10.1042/BSR20240482

**Published:** 2024-11-21

**Authors:** Aleena Das, Mrutyunjay Suar, K Sony Reddy

**Affiliations:** 1 School of Biotechnology, Kalinga Institute of Industrial Technology (Deemed University), Bhubaneswar, 751024, India; 2 Technology Business Incubator, Kalinga Institute of Industrial Technology (Deemed University), Bhubaneswar, 751024, India

**Keywords:** antimalarial, host-pathogen interactions, Human hormones, malaria, Plasmodium

## Abstract

Human malaria, caused by *Plasmodium* parasites, is a fatal disease that disrupts the host’s physiological balance and affects the neuroendocrine system. This review explores how malaria influences and is influenced by hormones. Malaria activates the Hypothalamus-Pituitary-Adrenal axis, leading to increased cortisol, aldosterone, and epinephrine. Cortisol, while reducing inflammation, aids parasite survival, whereas epinephrine helps manage hypoglycemia. The Hypothalamus-Pituitary-Gonad and Hypothalamus-Pituitary-Thyroid axes are also impacted, resulting in lower sex and thyroid hormone levels. Malaria disrupts the renin-angiotensin-aldosterone system (RAAS), causing higher angiotensin-II and aldosterone levels, contributing to edema, hyponatremia and hypertension. Malaria-induced anemia is exacerbated by increased hepcidin, which impairs iron absorption, reducing both iron availability for the parasite and red blood cell formation, despite elevated erythropoietin. Hypoglycemia is common due to decreased glucose production and hyperinsulinemia, although some cases show hyperglycemia due to stress hormones and inflammation. Hypocalcemia, and hypophosphatemia are associated with low Vitamin D3 and parathyroid hormone but high calcitonin. Hormones such as DHEA, melatonin, PTH, Vitamin D3, hepcidin, progesterone, and erythropoietin protects against malaria. Furthermore, synthetic analogs, receptor agonists and antagonists or mimics of hormones like DHEA, melatonin, serotonin, PTH, vitamin D3, estrogen, progesterone, angiotensin, and somatostatin are being explored as potential antimalarial treatments or adjunct therapies. Additionally, hormones like leptin and PCT are being studied as probable markers of malaria infection.

## Introduction

Malaria, caused by *Plasmodium* parasite, transmitted to humans through the bites of female *Anopheles* mosquitoes can be fatal. In 2022, approximately 249 million cases of malaria resulted in about 608,000 deaths [1]. The disease is characterized by fever, chills, headache, seizures, and difficulty in breathing, nausea, vomiting, and diarrhea [1]. If left untreated, malaria can cause severe complications and potentially lead to death [1].

Six malaria parasite species infect humans: *Plasmodium falciparum*, *P. vivax*, *P. ovale wallickeri*, *P. ovale curtisi*, *P. malariae*, and *P. knowlesi* [2]. *Plasmodium* infection in humans can lead to uncomplicated or severe malaria [2]. Severe malaria, responsible for the disease morbidity, is primarily caused by *P. falciparum* and, less frequently, by *P. vivax* and *P. knowlesi* [3]. The majority of malaria-related deaths are due to cerebral malaria, severe anemia, metabolic acidosis, and renal impairment [3]. Clinical symptoms of malaria like fever, anemia, and coma arise from the interplay between the parasite’s biology and the human body’s response [2,3]. The parasite’s ability to replicate within the host, combined with the host’s immune and physiological reaction, determines the extent of the disease’s severity [2].

Malarial infection disrupts host metabolism and results in hormonal imbalances. Research into hormonal responses to malaria reveals variations influenced by immune response, parasite species, infection severity, sex, age, nutritional status, and infection stage [4,5]. Understanding how these hormonal changes interact with the physiological effects of *Plasmodium* infection, including severe complications like cerebral malaria, is crucial. This knowledge may help develop strategies to improve preparedness and reduce the malaria burden.

**Figure 1:**
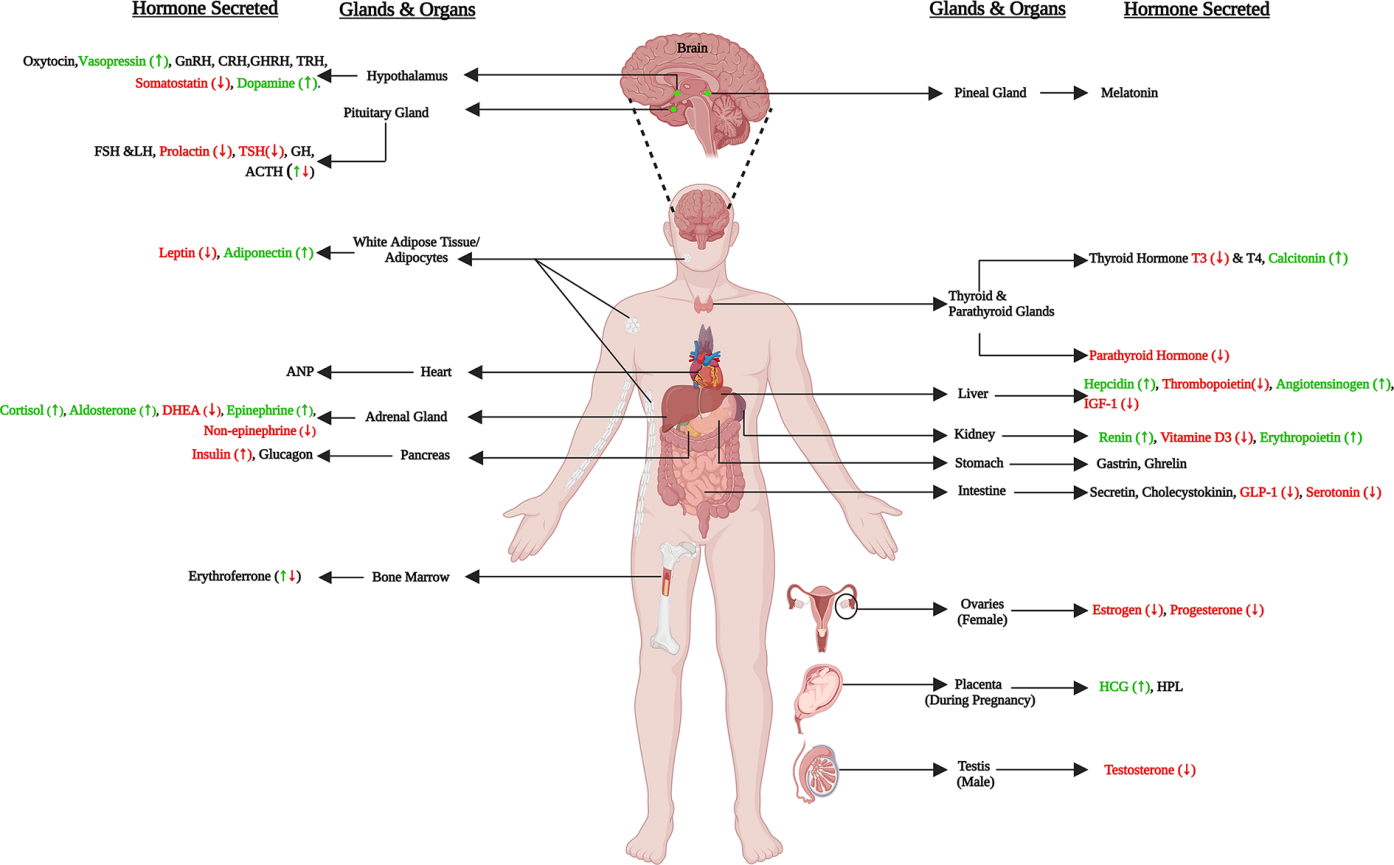
Human endocrine organs and the major hormones produced For hormones, where dysregulation has been reported during malaria has been marked by up (green) and down (red) arrows.

Hormones are chemical messengers that are secreted into the bloodstream, majorly by the endocrine glands (Figure 1) [6]. They travel to various tissues and organs, where they bind to specific receptors and elicit responses, helping to maintain homeostasis and regulate a wide range of physiological processes [6]. Hormones play a crucial role in regulating various body functions, including immune responses, growth, metabolism, and response to infection [6,7]. For example, insulin and glucagon, produced by the pancreas, are responsible for blood glucose homeostasis [6,7]. Estrogen and testosterone are responsible for reproductive functions, while hepcidin, synthesized in the liver, is involved in iron homeostasis [6,7].

Research on hormone dysregulation related to *Plasmodium* infection has been extensive, but no comprehensive summary exists of the collective hormonal responses across different hosts. This review seeks to elucidate the complex interactions between *Plasmodium* infection and hormonal regulation in humans and various animal models. We have organized hormones into organ-specific groups to clarify host-parasite interactions, disease mechanisms, and potential therapeutic targets (Figure 1). By linking these hormonal changes to their physiological effects, we aim to offer insights that could be used to enhance health and well-being for those affected by malaria.

## Hypothalamus

**Figure 2:**
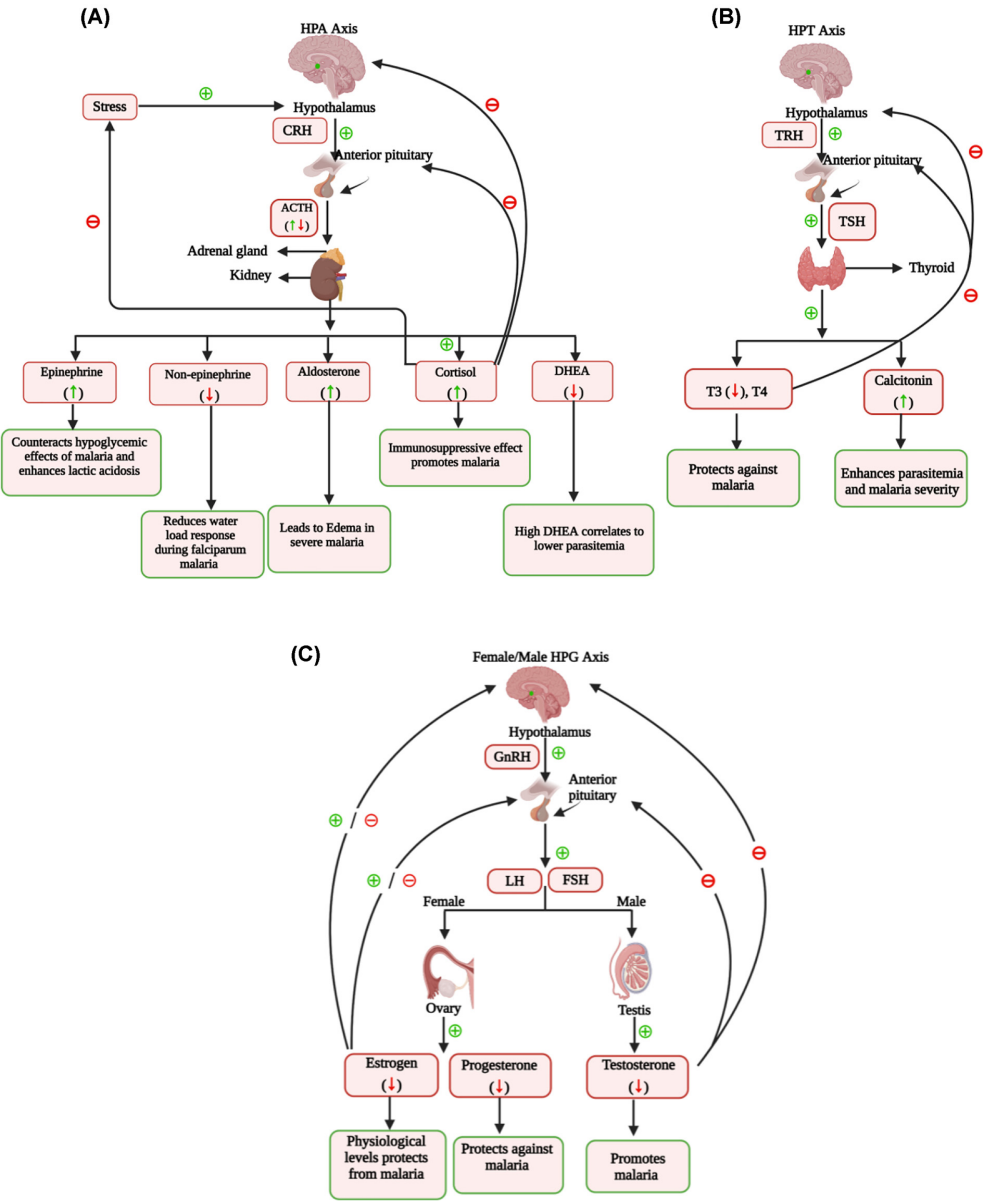
The impact of malaria on Hypothalamus-Pituitary-Adrenal (HPA), Hypothalamus-Pituitary-Thyroid (HPT) and Hypothalamus-Pituitary-Gonad (HPG) axis Visual representation of the various organs and hormones involved in the (**A**) HPA, (**B**) HPT, and (**C**) HPG hormonal axis. The hormones upregulated during malaria are marked by up arrows; whereas those downregulated are denoted by down arrows. The effect of the hormones on the malaria parasite or pathology are highlighted in the green-outlined boxes.

The hypothalamus, a small region within brain, acts as a crucial link between the nervous and endocrine systems [6,7]. Hypothalamic hormones produced through neurons, get influenced by external and internal environments, as well as hormonal feedback [6,7]. Hypothalamic hormones released into the hypothalamic-hypophyseal portal system, majorly regulate pituitary hormone release, which in turn stimulate hormone release from the target glands like adrenal, thyroid and gonads [6,7] (Figures 1 and 2). This regulatory interplay is exemplified by the hypothalamic-pituitary-adrenal (HPA) axis, the hypothalamic-pituitary-thyroid (HPT) axis, and the hypothalamic-pituitary-gonadal (HPG) axis, that gets dysregulated during malaria (Figure 2) [4,6].

### Posterior pituitary regulating (neurohypophysis) hormones

#### Oxytocin

Oxytocin is a neuropeptide involved in stress regulation, social bonding, birth, lactation, and immune function [6–8]. It reduces inflammation by inhibiting microglia and macrophage responses and decreasing pro-inflammatory cytokines like TNF-α and IL-1β [8]. The role of oxytocin in immunomodulation, neuroprotection warrants active research to understand oxytocin level and its effect on physiology during cerebral and placental malaria [8].

#### Anti-diuretic hormone (ADH) or vasopressin

A peptide hormone that plays a crucial role in regulating water retention (water re-absorption from kidneys) and blood pressure (vasoconstriction) in the body [6]. In malaria patients with hyponatremia, arginine and vasopressin levels were enhanced [9].

### Anterior pituitary affecting hormones

#### Thyrotropin releasing hormone (TRH)

TRH, a tri-peptide hormone synthesized in the hypothalamus’s paraventricular nucleus, is processed from pro-TRH to its active form [7]. It primarily stimulates the release of thyroid stimulating hormone (TSH) and prolactin from the anterior pituitary, impacting key physiological processes [7]. While levels of thyroid hormones like T3, thyrotrophin, and thyroxine-binding protein are reported to be lower during malaria, no such report exists for TRH [10].

#### Somatostatin (growth hormone-inhibiting hormone)

Somatostatin is a peptide hormone that regulates the endocrine system, neurotransmission, and cell growth by binding to G-protein coupled receptors [7]. It is abundant in the nervous system, pancreas, and gastrointestinal tract [7]. It inhibits hormone release, including insulin, glucagon, growth hormone, TSH, and prolactin, and slows gastric emptying, intestinal contractions, and pancreatic secretion [7].

Hyperinsulinemia (insulin is a hormone that reduces blood glucose levels [7]), is common during *P. falciparum* malaria [11]. Somatostatin inhibits insulin release [7], and thus its analogue sandostatin can prevent hyperinsulinemia induced hypoglycemia in *P. falciparum* malaria [11]. Reduced somatostatin levels are seen in the brain cortex of mice with cerebral malaria [12]. The antimalarial drug artemether has been shown to increase somatostatin levels in pancreatic islets (Table 1) [13].

**Table 1:** List of antimalarial drugs influencing the human hormones or their receptors

S. No.	Antimalarial name	Affected hormone/organ	Noted alterations
**1**	Artemisinin/artemisinin derivatives	Somatostatin	Artemether enhance somatostatin levels in pancreatic islets.
		Testosterone	Artesunate reduces testosterone levels in male guinea pigs.
		Thyroid/thyroid hormone	Artemisinin enhances thyroid function and hormone production.
		Calcitonin	Artemisinin and dihydro-artemisinin stop calcitonin receptor activation.
		Pancreas/insulin	Artesunate prevent Type-1 diabetes in mice by reducing autoimmune T cells and enhancing protective T cells.
**2**	Piperaquine	ACTH	Reduces ACTH secretion.
**3**	Chloroquine	Aldosterone	Reduces aldosterone levels.
		Vasopressin	Reduces vasopressin levels.
		Testosterone	Reduces testosterone levels in rat.
		Erythropoietin	Chloroquine reduces erythropoietin levels in healthy individuals but enhances it in malaria patients.
		Serotonin	Act as antagonist for 5-HT3.
**4**	Hydroxychloroquine	Insulin	Improves insulin sensitivity.
		Adiponectin	Enhances adiponectin levels.
**5**	Quinine	Insulin	Increase insulin levels.
		Serotonin	Decrease serotonin secretion; act as antagonists to 5-HT3.
**6**	Mefloquine	Serotonin	Acts as partial 5-HT2A agonist, full 5-HT2C agonist, 5-HT3 antagonist.

### Dopamine

A catecholamine and neurotransmitter secreted by hypothalamus, also acts as a hormone to regulate the pituitary and inhibit the secretion of prolactin [7]. Disrupted dopamine receptor signaling significantly contributes to the dysfunction of striatal neurons in experimental cerebral malaria (ECM) [14]. Dopamine has also been reported to activate the enzymatic activity of *P. falciparum* carbonic anhydrase [15]. Dopamine levels get enhanced in the brain of mice with ECM [16].

Other relevant peptide hormones secreted by the hypothalamus include gonadotropin-releasing hormone (GnRH), which stimulates the pituitary to release gonadotropins; corticotropin-releasing hormone (CRH), which activates the HPA axis to prompt ACTH release; and growth hormone-releasing hormone (GHRH), which triggers the release of growth hormone and promotes somatotroph proliferation [6,7]. However, there are no reports investigating the effects of these hormones on malaria pathology.

## Pituitary

Located below the hypothalamus, the anterior pituitary produces adrenocorticotrophic hormone (ACTH), gonadotropins (FSH and LH), TSH, growth hormone, and prolactin (Figures 1 and 2) [6,7]. The posterior pituitary stores and releases vasopressin and oxytocin, synthesized by hypothalamic neurons (Figure 1) [6,7].

### Follicle-stimulating hormone (FSH) & Luteinizing hormone (LH)

FH and LH are glycoprotein hormones crucial for reproduction [7]. In women, FSH promotes ovarian follicle growth and estrogen production, while LH triggers ovulation and progesterone release [7]. In men, FSH supports sperm production and LH stimulates testosterone production (Figure 2) [7]. Antimalarial drugs like artesunate and ACTs have shown no impact on serum FSH and LH in rats and guinea pigs, though they reduced testosterone levels in male guinea pigs (Table 1) [17–19].

### Prolactin

A peptide hormone that regulates milk production, reproductive health and also affects the immune system and behavior [7]. Prolactin stimulates the cytotoxic capability of NK cell [20]. Lower prolactin levels in infected females [21] suggest it plays a role in malaria, particularly in placental malaria [22]. However, some argue that increased cortisol, not prolactin, reduces NK cytotoxicity and heightens malaria risk in pregnant women [20].

### Thyroid stimulating hormone (TSH)

TSH regulates thyroid gland activity, influencing metabolism, energy levels, and overall growth [7]. Children with uncomplicated falciparum malaria have lower TSH levels [23].

### Growth hormone (GH)

GH is essential for growth, metabolism, and cell repair, with roles in immunomodulation [7,8]. It raises blood glucose by reducing glucose uptake and increasing liver gluconeogenesis, while enhancing amino acid uptake, protein synthesis, and lipid breakdown [7]. GH influences the immune system by enhancing T cell development, cytokine production, B cell activity, and neutrophil and monocyte function, while also promoting cell adhesion and anti-apoptotic actions [24]. GH stimulates the IGF-1 hormone production in the liver and kidneys [7]. Malaria affects growth, metabolism, and causes anemia [2,3]; and thus, impact of malaria on GH should be investigated.

### Adrenocorticotrophic hormone (ACTH)

ACTH, regulates the adrenal cortex’s release of corticosteroids, impacting stress response and metabolism [7]. ACTH levels were found to be heterogeneous in malaria patients [25]. Tetracosactrin, a synthetic ACTH analogue, increased plasma corticosterone levels, and in those (Swiss mice) with established immunity to *Plasmodium berghei*, this rise correlated with a loss of immunity to the malaria parasite [26]. ACTH administered to malaria patients leads to increase in parasitemia [27]. Antimalarial drugs like piperaquine, and artemisinin-piperaquine combination reduce ACTH secretion in rats (Table 1) [19]. Although ACTH is not directly involved in malaria, its role in cortisol regulation [7] may affect malaria severity due to cortisol’s immunosuppressive effects [20] (Figure 2).

## Adrenal glands

The adrenal glands, situated atop each kidney [6]. The adrenal cortex produces steroid hormones like cortisol, aldosterone, and adrenal androgens, essential for stress response, electrolyte balance, and metabolism (Figure 1) [6,7]. Adrenal medulla produces adrenaline and noradrenaline (Figure 1) responsible for flight or fight response [6].

Adrenalectomy in mice results in early onset of severe hypoglycemia and heightened inflammation during malaria [28]. Adrenal hormones enhance malaria resistance by preventing severe hypoglycemia and excessive systemic and brain inflammation [28].

### Cortisol

Cortisol, the ‘stress hormone,’ regulates stress responses, metabolism, and immune functions [6,7,20]. Elevated levels are observed in *P. falciparum* and *P. vivax* malaria [28–35], as well as in pregnant women and malnourished children [36]. Pregnant women, especially primigravidae show significantly higher cortisol levels, which contributes to increased malaria susceptibility [21,30,37,38]. Similar to humans, malaria-infected mice show higher cortisol levels [39].

Cortisol’s immunosuppressive effects include reducing the pro-inflammatory cytokine production and inhibiting NK cell activity [20,25,30,40]. NK cells help combat malaria in the pre-erythrocytic and erythrocytic stages, and their absence in intervillous tissue is linked to increased susceptibility to human placental malaria [30,41]. Elevated cortisol also aids in gluconeogenesis, potentially mitigating malaria-induced hypoglycemia in malaria patients [42]. Cortisol may also affect expression of human genes involved in antimalarial drug metabolism, potentially altering treatment efficacy [43].

Hydro-corticosterone administration increases murine malaria susceptibility by suppressing the immune system [44,45]. However, Liposome-encapsulated β-methasone hemisuccinate (prodrug form of the glucocorticoid β-methasone) also prevents cerebral malaria and reduces edema, hemorrhages, and inflammation (Table 2) [46,47]. Dexamethasone, a synthetic glucocorticoid, can counteract adrenalectomy effects, prevent hypoglycemia, reduce IL-17 cytokine levels, and improve survival in infected mice by inducing anti-inflammatory effects [28,48]. Early dexamethasone treatment in mice prevents lethal cerebral malaria and reduce organ damage and inflammation (Table 2) [25,49]. In a murine MA-ARDS (malaria-associated acute respiratory distress syndrome), high doses of dexamethasone can mitigate lung pathology, accompanied by reduced pulmonary inflammation [25,50]. Inhibition of brain iNOS (inducible nitric oxide synthase) by Dexamethasone leads to lower parasitemia and enhanced survival of *P. berghei* infected mice [51]. Dexamethasone was speculated to benefit patients with cerebral malaria [52], but later was shown to be deleterious in comatose patients with cerebral malaria [53,54].

**Table 2:** List of hormones explored for development of antimalarial therapy

S. No.	Hormone name	Molecules with antimalarial activity
**1**	Cortisol	Liposome-encapsulated β-methasone hemisuccinate prevents cerebral malaria, edema, hemorrhages, and inflammation.
		Dexamethasone, reduces ECM, organ damage and inflammation; thereby enhancing survival of mice with malaria. Failed preclinical trial as adjunct therapy in malaria.
**2**	DHEA	High serum DHEA prevent malaria and intraerythrocytic growth of parasites.
		DHEA analogue- 16α-Bromoepiandrosterone, enhances phagocytosis ring-stage infected erythrocytes.
**3**	Estrogen	Physiological levels of estrogen prevent malaria.
		Selective estrogen receptor modulators (SERMs) like tamoxifen, raloxifene, and bazedoxifene exhibit antimalarial properties.
		Artemisinin-estrogen hybrids, have been reported to inhibit the growth of *P. falciparum*.
**4**	Progesterone	Progesterone and its analogs inhibit growth of *P. falciparum*.
**5**	Melatonin	Luzindole (Melatonin receptor antagonist), hampers intraerythrocytic *P. falciparum* parasite growth.
		Melatonin treatment enhances survival in mice with malaria.
		Triazine-indole and indole alkaloids (melatonin indole derivatives) display antiplasmodial activity. Cipargamin in clinical trials.
**6**	1,25-Dihydroxyvitamin D3	Calcitriol and 22-oxacalcitriol display antiplasmodial activity against *P. falciparum* and *P. chaubadi*.
		Arteether and Vitamin D3 combination improves ECM survival.
**7**	Erythropoietin	Artesunate-erythropoietin treatment induces early recovery from *P. bergei* induced murine malaria.
		Erythropoietin combined with quinine have been reported safe in short-term malaria treatment.
**8**	Hepcidin	Exogeneous administration protects mice from ECM.
**9**	Angiotensin-II	AT-1 receptor blockers like irbesartan and losartan improve survival in cerebral malaria.
		Angiotensin II derivatives like VIPF and Ang II-SS protect against severe malaria without vasoconstriction.
**10**	Serotonin	4-Methoxymeridianin A, and 20 -debromo-20-chloro analog of psammopemmin- that could bind with the serotonin receptors, inhibit *P. falciparum* growth in vitro.
		Dihydroergotamine methanesulfonate (serotonin receptor antagonist) inhibit *P. falciparum* growth in vitro.
		Serotonin receptor agonists like 8-OH-DPAT reduces *P. falciparum* growth in vitro in strain transcending manner.
		6-bromoaplysinopsin, ligand of serotonin receptor 5-HT2, displays antiplasmodial activity.
		TCMDC-139046, which interacts with serotonin antagonist receptors 5-HT2, displays antimalarial efficacy.
		Citalopram, a 5-HT reuptake inhibitor reverses the chloroquine resistance of *P. falciparum* and *P. chaubadi*.
		p-chlorophenylalanine (serotonin synthesis inhibitor) and cyproheptadine (serotonin, bradykinin and histamine antagonist) reduce the parasitemia for *P. yoelii nigeriensis*-induced malaria.

### Aldosterone

**Figure 3:**
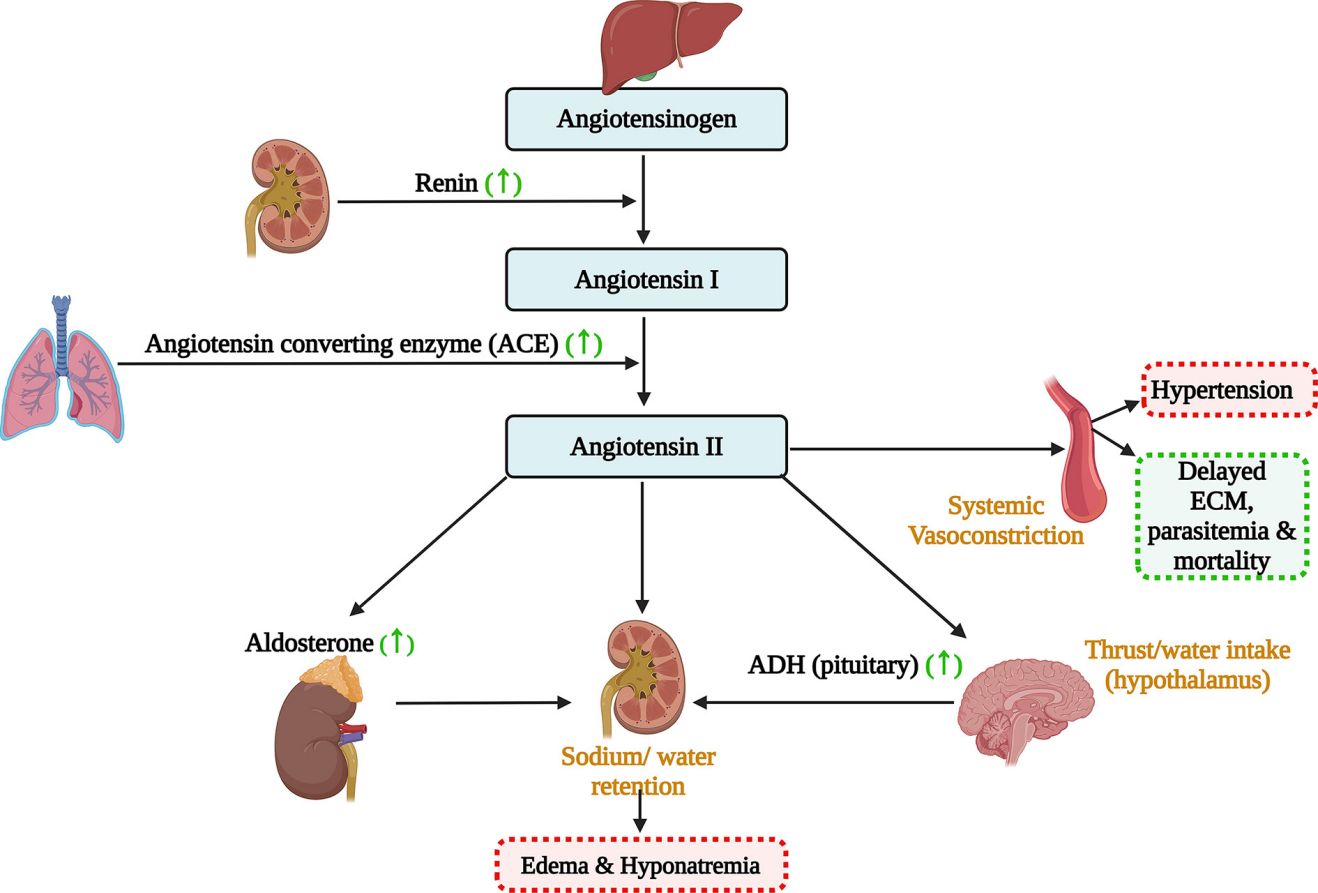
Diagrammatic overview of the Renin-Angiotensin-Aldosterone System (RAAS) and its role in malaria pathology The hormones or enzymes upregulated has been denoted by upward arrows. Higher levels of angiotensin-II, leads to vasoconstriction, whereas higher levels of aldosterone and anti-diuretic hormone (ADH) leads to edema and hyponatremia.

Aldosterone, a mineralocorticoid secreted by the adrenal cortex’s zona glomerulosa, regulates electrolyte balance by promoting sodium reabsorption and potassium excretion, essential for blood pressure and fluid volume [6,55]. It plays a key role in the renin-angiotensin-aldosterone system (RAAS) (Figure 3), crucial for controlling renal, cardiac, and vascular functions [55].

Severe *falciparum* malaria is associated with hyponatremia, increased urinary aldosterone, and altered sodium-potassium ratios [56,57]. High aldosterone can lead to sodium retention and edema- a common symptom in severe malaria cases [57]. Chronic chloroquine use has been linked to increased aldosterone and vasopressin levels (Table 1), but also to kidney injury, reducing blood pressure, glomerular filtration rate, and urine output [58]. Aldosterone activates immune cells, stimulates proinflammatory factors and the production of adhesion molecules and cytokines, and thus, may play an important role against malaria pathogenesis [59].

### Adrenal Androgens

Dehydroepiandrosterone (DHEA) and its sulfate form (DHEAS) are precursors to sex hormones and play roles in immune modulation, stress response, and inflammation regulation [60]. DHEA counters cortisol’s immunosuppressive effects by boosting anti-inflammatory cytokine production and immune cells like NK cells, neutrophils, and macrophages [60].

Low DHEA levels are linked to weakened immunity and higher infection rates [60]. High serum DHEA/DHEA-S levels correlate with lower parasite density in malaria-infected individuals (Figure 2 and Table 2) [29,61,62]. DHEA non-competitively inhibits Glucose-6-phosphate dehydrogenase (G6PD) [63,64], thus reducing NADPH production. NADPH supports glutathione levels and protects red blood cells from oxidative damage [64]. G6PD deficiency disrupts this balance, preventing malaria parasite growth in erythrocytes [64,65]. Thus, DHEA inhibits the intraerythrocytic growth of the human malaria parasite- *P. falciparum* tested in vitro, and of the murine malarial parasite- *P. berghei* tested in vivo [63,65,66]. A DHEA analogue- 16α-Bromoepiandrosterone, has been reported to enhance phagocytosis of ring-stage infected erythrocytes tested in vitro (Table 2) [67].

### Epinephrine

Epinephrine or adrenaline, is a neurotransmitter that plays an imperative role in the ‘fight or flight’ response [7]. It is commonly used in medicine to treat severe allergic reactions (anaphylaxis), asthma attacks, and certain cardiac conditions [7]. Adrenaline injection has been reported to lower insulin concentration and reduce peripheral parasitemia in certain murine strains, thus reversing hypoglycemic effect during murine malaria [68,69]. Enhanced brain epinephrine levels have been reported in *P. berghei* infected male and female C57Bl/6 mice [16]. Epinephrine infusions have been reported to increase lactic acidosis in plasma of infected patients (Figure 2) [70].

### Norepinephrine

Norepinephrine, a key player in the ‘fight or flight’ response, affects heart rate, blood vessel constriction, and energy mobilization [7]. In severe falciparum malaria, children show reduced production of norepinephrine, though histamine is elevated [71]. Additionally, falciparum malaria patients with reduced water load-response display elevated plasma norepinephrine levels (Figure 2) [72]. During *P. berghei* induced murine malaria, norepinephrine levels were lower in the brain of infected females whereas enhanced in the brain of infected males [16].

## Thyroid

The thyroid gland, located in front of the trachea below the larynx, has two lobes and produces thyroid hormones [thyroxine (T4) and triiodothyronine (T3)], and calcitonin [6,7] (Figure 1).

Acute severe falciparum malaria suppresses thyroid function [73]. Conversely, the antimalarial drug artemisinin enhances thyroid function and hormone production (Table 1) [74]. Hypothyroidism has been reported to protect mice from *P. berghei* induced ECM, but does not reduce parasite burden nor rescue mice from death [75].

### T3 (Tri-iodothyronine), T4 (Thyroxine)

Thyroid hormone (T3 and T4) boosts metabolism in nearly all body tissues, aids in heat production, and supports energy generation from carbohydrates, proteins, and lipids. T3 is more active and has three iodine atoms, while T4, with four iodine atoms, serves as a precursor to T3 [6,7].

Reduced T4 production, induced by 6-propylthiouracil (6-PT), resulted in lower parasitemia in *P. berghei* infected mice, suggesting thyroid hormones may influence malaria infection [76]. In severe falciparum malaria, TSH levels stay the same, prolactin slightly increases, T3 levels drop, and T4 remains stable or increases (Figure 2) [10]. Another recent study on children with uncomplicated falciparum malaria reported lower TSH, elevated T3, with stable T4 levels [23]. This suggests thyroid hormones may affect malaria severity, but the exact mechanism is unclear.

### Calcitonin (CT)

Calcitonin, a 32-amino acid peptide hormone secreted by thyroid parafollicular cells (C cells), lowers blood calcium levels, opposing parathyroid hormone. Initially synthesized as 141-amino acid pre-ProCT, gets cleaved at the amino-terminus to form the 116-amino acid procalcitonin (PCT), which is then further processed to CT [77]. CT, regulated by serum calcium and gastrin, is crucial for bone resorption and skeletal homeostasis [77]. It also decreases phosphate and calcium reabsorption in the kidneys and can lower testosterone, LH, and FSH levels [77]. Chronic CT use in migraine patients increases β-endorphin, ACTH, and cortisol levels, and high CT levels in the gastrointestinal tract promote water and electrolyte release [77].

Higher PCT levels has also been reported in patients with severe [78,79] and uncomplicated malaria [80] with serum PCT levels being suggested to be used as an important marker to detect *P. falciparum* malaria [81–83]. However higher PCT levels are also observed in case of bacterial infection, thus limiting the notion to use PCT levels as diagnostic marker for malaria [84,85]. Calcitonin has been shown to enhance parasitemia, anaemia, and reticulocyte count in mice infected with *P. chabaudi* [86]. The antimalarial drug- artemisinin and dihydroartemisinin has been reported to stop calcitonin receptor activation (Table 1) [87,88].

## Parathyroid

The parathyroid glands are four tiny, pea-sized structures located behind the thyroid that produce parathyroid hormone [6].

### Parathyroid hormone

Parathyroid hormone (PTH) is crucial for regulating calcium and phosphorus metabolism, maintaining bone health, and supporting various physiological functions [89]. In *P. falciparum* malaria, patients often exhibit hypocalcemia, hypophosphatemia, and low PTH levels [34,90], with parathyroid gland failure potentially contributing to these abnormalities [91]. Since calcium is vital for the survival and replication of *Plasmodium* parasites, changes in PTH levels could affect calcium availability and influence malaria severity [91].

PTH has immunomodulatory effects [89]. In murine malaria, prolonged PTH treatment has been shown to prevent infection-induced proliferation of hematopoietic stem cells (HSCs), partially preserve osteoblasts, and lower global IFN-γ levels by reducing the number of IFN-γ-secreting T cells [92]. PTH, combined with ROS quenching, also partially restores hematopoietic stem cell (HSC) function [92]. Given PTH’s impact on HSC, variations in PTH levels could affect erythrocyte production and may influence malaria-associated anemia severity [92].

## Gonads

The gonads (ovaries and testes) produce germ cells and synthesize steroid sex hormones (Figures 1 and 2) [6,7]. These hormones are crucial for developing reproductive organs, secondary sex characteristics, and processes like pregnancy and lactation [6,7]. There are three main hormones: estrogens for feminizing effects, progestogens (e.g., progesterone) for uterine preparation during pregnancy, and testosterone for masculinizing effects [6,7]. Beyond reproduction, sex hormones also impact carbohydrate and lipid metabolism, cardiovascular health, and bone growth [6].

### Estrogens

Estrogen is a sex hormone found at higher levels in females but also in males in smaller amounts [6,7]. It regulates various physiological processes, including the regulation of the immune system [5]. Lower levels of estradiol have been reported in patients with severe falciparum malaria (Figure 2) [93]. Female susceptibility to malaria can vary from males, potentially influenced by estrogen [94,95]. Female mice exhibit higher oxidative stress and parasitemia in response to *P. berghei* infection, suggesting estrogen’s influence on oxidative stress [94,95]. Castrated female BALB/c mice succumb to *P. berghei* induced malaria more rapidly than controls, but those treated with estradiol have improved survival [96]. Estrogen administration exacerbates *P. chabaudi* induced infection severity in mice, while reducing parasitemia in gonadectomized females [97]. Although estradiol can decrease parasitemia, it worsens cerebral malaria and increase mortality in *P. berghei*-infected mice [98].

Estrogen can impact immune function by altering antibody production and immune cell activation, influencing the response to *Plasmodium* infection [99]. 17β-estradiol has been shown to induce sex-specific variations in parasitemia, body mass, temperature, hemoglobin levels, CD8+ T cells, and NK1.1+ cells in the spleen [100]. It also alters mRNA expression of Tnf and Il1b in the brains of *P. berghei*-infected mice and plasma cytokine levels [100]. Physiological levels of estrogen have been suggested to enhance immunity and protect female mice from *P. chaubadi*-induced disease symptoms (Table 2) [97,101]. However, higher estrogen levels have been reported to be immunosuppressive and suppress self-healing of *P. chaubadi*-mediated malaria in C57BL/10 mice [102].

During pregnancy, hormonal changes, including increased estrogen levels, are significant [103]. This hormonal shift may contribute to the heightened susceptibility of pregnant women to malaria [103]. Pregnant women are known to be more susceptible to malaria infection, and estrogen could be one of the factors contributing to this increased vulnerability [103]. Lower 17β estradiol levels have been reported in serum of women with non-placental malaria [93]; whereas higher levels have been reported in placental blood of women with placental malaria [104].

Selective estrogen receptor modulators (SERMs) like tamoxifen, raloxifene, and bazedoxifene exhibit antimalarial properties (Table 2), with bazedoxifene showing potent efficacy [99,105,106]. Bazedoxifene inhibited in vitro growth of *P. falciparum* from both male and female origin and demonstrated antimalarial activity in *P. berghei*-infected female BALB/c mice but not in males [106]. In contrast, treating *P. berghei* -infected female CBA/Ca mice with tamoxifen resulted in an increased parasite load, exacerbated symptoms by reducing body temperature and body weight, and worsened anemia [99]. Additionally, artemisinin-estrogen hybrids, have been reported to inhibit the in vitro growth of *P. falciparum* with efficacy similar to dihydroartemisinin (Table 2) [107]. Estradiol chalcone derivatives have also been reported with mild antimalarial effects in in vitro tests against *P. falciparum* [108].

### Progesterone

It is produced by the ovaries in women, as well as by the placenta during pregnancy [103,109,110]. It regulates the menstrual cycle, prepares the uterus for pregnancy, and supports early pregnancy by maintaining the uterine lining [7].

Progesterone may have a protective effect against malaria [93,101,111]. Serum levels of progesterone along with cholesterol, estrogen, testosterone, and Vitamin D have been reported to be lower in patients with *P. falciparum* malaria [93,111]. Low serum progesterone levels were also reported in murine malaria [101]. Progesterone and its analogs have been reported with growth inhibitory activity against *P. falciparum* tested in vitro (Table 2) [112]. Also, steroid hormones (17-beta-estradiol, progesterone, and testosterone) have been reported to increase the gametocytogenesis of *P. falciparum* in vitro [113]. During humanplacental malaria, IL-7 and IFN-γ have been suggested to improve pregnancy outcomes by maintaining plasma levels of progesterone, maternal hemoglobin, and HDL-C [104].

### Testosterone

Testosterone, primarily a male hormone, also exists in smaller amounts in females and is linked to male development and reproductive functions [7]. Low levels of testosterone have been reported in patients with *P. falciparum* malaria (Figure 2) [93,114], with testosterone levels being positively correlated with parasitemia caused by *P. vivax* in adult males [35]. The antimalarial drug chloroquine has been reported to reduce fetal testosterone levels and testis development in rat (Table 1) [115].

*P. berghei*-infected albino male mice exhibit low serum testosterone and high cortisol levels [39]. Exogenous testosterone affects infection differently based on sex: it increases parasitemia in male mice but decreases it in females [116]. Testosterone exacerbates *P. berghei* induced malaria pathogenesis in male mice by increasing CD8^+^ cells, reducing Mac3^+^ macrophages, and suppressing IL-17A, which is linked to anemia [117]. During murine malaria infection, testosterone impact induces a substantial decrease in the mRNA levels of the malaria-responsive gene, lowered IFN-γ and decreased regulatory T-cell mRNA expression during the peak of parasitemia [109,110,118,119].

Studies suggest that the liver mediates testosterone’s suppression of malaria protection [120]. Testosterone affects liver metabolism, dampens immune responses, and may contribute to hepatocellular carcinoma [121]. Testosterone disrupts antimalaria defenses in the spleen and liver, potentially leading to fatal outcomes in malaria-resistant mice [122]. It alters lincRNA and mRNA expression in the spleen, impacting malaria defense [122]. Testosterone-induced lethal outcomes during blood-stage malaria occur exclusively in naïve mice, while immune mice are unaffected by testosterone’s effects [123]. The liver’s role has been proposed for the acquired antibody-mediated protection against blood-stage malaria, which happens through selective unresponsiveness to testosterone-induced gene expression [124]. Testosterone treatment reduces the mice’s spleen size and the total number of spleen cells, with the adoptively transferred splenic T-cells being able to suppress the self-healing of C57BL/10 mice against *P. chaubadi* malaria [125]. During blood-*stage P. chabaudi* infections, mRNA levels of TLR1, TLR2, TLR4, TLR6, TLR7, and TLR8 increase in hepatocytes [126]. Testosterone pre-treatment amplifies TLR6 expression 5.6-fold and suppresses TLR8 expression 6.5-fold, suggesting dysregulated TLR6 and TLR8 signaling contributes to increased malaria susceptibility [126].

## Placenta

The placenta is a crucial feto-maternal organ that secretes hormones to sustain pregnancy, supports lactation, and manages nutrient and waste exchange between mother and fetus [127,128]. The placenta produces steroid hormones like progesterone and estrogens, and peptide hormones such as human chorionic prolactin and human placental lactogen (Figure 1) [127,128]. It also releases other peptide hormones like human chorionic thyrotrophin, human chorionic adrenocorticotrophic hormone, placental growth hormone, PYY, calcitonin gene-related peptide, prolactin-releasing peptide, activin, follistatin, inhibin, urocortin, and leptin, though their precise physiological functions are not yet fully understood [128].

### Human Chorionic gonadotropin (HCG)

HCG maintains the corpus luteum, supports fetal development, and is used in pregnancy tests [128]. It aids fertility treatments, regulates maternal metabolism, and modulates immune tolerance. HCG also promotes *P. falciparum* growth in vitro, potentially worsening malaria severity in pregnancy [129].

### Human somatomammotropin/Human Placental Lactogen (hPL)

Somatomammotropin regulates maternal metabolism, supports fetal growth, and prepares the breasts for lactation [128]. It increases maternal insulin resistance to provide glucose for the fetus and may indicate complications like gestational diabetes [128]. hPL also promotes maternal immune tolerance to the fetus [127].

During pregnancy, malaria infection can result in placental malaria, in which *P. falciparum*-infected red blood cells bind to placental receptors [130]. This adhesion triggers inflammation and damage to the placenta, adversely affecting both the mother and the infant [130]. Though placental malaria is a major complication [2,3], studies linking hormones to placental malaria are lacking and would need urgent attention.

## Pancreas

The pancreas, located in the abdomen has both exocrine and endocrine functions [6,7]. The endocrine function includes secretion of insulin and glucagon from the pancreatic islet of Langerhans (Figure 1) [6].

### Insulin and glucagon

Insulin and glucagon are key regulators of blood glucose levels [6,7]. Produced by the pancreas, insulin is released by beta cells and lowers blood glucose by facilitating cellular uptake, promoting glycogen and fat storage, supporting protein synthesis, and inhibiting glucose production and breakdown in the liver [6,7]. Glucagon, secreted by alpha cells, raises blood glucose, especially during fasting, by stimulating glycogen breakdown and glucose production in the liver, inhibiting glucose use, and promoting fat breakdown [6,7]. Together, these hormones maintain glucose homeostasis and overall metabolic balance [6,7].

Diabetes affects the immune system, healing, and metabolism, promoting parasite infection spread [131]. In uncomplicated malaria, stress hormones and cytokines can induce insulin resistance, raising the risk of developing type 2 diabetes [132,133]. Placental malaria can impair insulin production, resulting in elevated blood glucose levels [134]. Additionally, fetal exposure to placental malaria, reduces birth weight, and may enhance risk of type 2 diabetes in early adulthood [135].

In severe malaria, glucose production can be impaired, leading to hypoglycemia. Hypoglycemic patients with *P. falciparum* malaria often show increased insulin secretion and enlarged pancreatic islets [136]. Conversely, elevated insulin levels (hyperinsulinemia), a major cause of hypoglycemia, are frequently seen in cases of severe malarial hypotension [137]. Severe hypoglycemia and hyperinsulinemia have also been observed in murine infections with both non-lethal *P. chabaudi* and lethal *P. yoelii* strains [138].

High blood sugar (hyperglycemia) is also an occurrence in severe malaria (SM) [139] with a stronger association seen in cases of cerebral malaria (CM) [137], where enhanced glucose production and gluconeogenesis has been reported [140–142]. Children suffering from malaria displayed elevated basal glucose levels and increased endogenous glucose production compared to their healthy counterparts [143,144].

Insulin treatment improves survival in mice infected with *P. berghei*, indicating its potential as an additional therapy for malaria [145]. This treatment reduces pro-inflammatory cytokines (TNF-α and IFN-γ) while enhancing anti-inflammatory cytokines (IL-4 and IL-10) [145]. Insulin’s impact on cytokines is linked to its ability to inhibit GSK3β, leading to the suppression of NF-κB p65 activation [145]. On contrary, insulin enhances blood stage *P. falciparum* infection in vitro [131]. In addition, malaria parasites can also use insulin to their advantage [131]. Insulin receptors on red blood cells, on being activated increases glucose uptake inside infected erythrocyte, thus promoting parasite growth [131].

The antimalarial drug Hydroxychloroquine has been reported with anti-diabetic effect [146]. Hydroxychloroquine’s antidiabetic effect may involve reduced lysosomal degradation of the insulin-receptor complex, improved insulin sensitivity, and increased adiponectin levels (Table 1) [146]. Artesunate can prevent T1D in NOD mice by decreasing autoimmune T cells and raising protective T cells (Table 1) [147]. Chloroquine exhibits promising potential as a therapeutic agent for individuals with type 2 diabetes mellitus by virtue of its innovative role as an activator of Akt, which subsequently enhances glucose uptake and glycogen synthase activity [148]. Quinine administration in malaria patients, enhances the blood insulin levels resulting to decline in serum glucose concentration (Table 1) [68,140,141,149–151].

## Pineal Gland

The pineal gland, located in the brain’s posterior cranial fossa, regulates sleep-wake cycles and is also known as the epiphysis cerebri [152]. It contains pinealocytes and supporting cells, producing key hormone like melatonin from tryptophan [152].

### Melatonin

Melatonin, derived from tryptophan, regulates the sleep-wake cycle, acts as an antioxidant, and influences immune function, mood, and reproduction [7]. Melatonin modulates various aspects of the malaria infection [154–170]. It affects malaria by synchronizing parasite stages, as seen with *P. falciparum*, which transitions to the schizont stage faster when cultured with melatonin [153,154]. Likewise, *P. chabaudi* parasites grew non-synchronous in mice without pineal glands or administered with melatonin receptor antagonist luzindole, but melatonin restored it [154]. However, melatonin does not synchronize *P. berghei* or *P. yoelii* in vitro [155], and disruptions in rhythmic patterns of the host and parasite, impact parasite growth and transmission efficiency [156,157].

Melatonin influences the intraerythrocytic cycle of *P. falciparum* by activating the PLC-IP3 signaling cascade [158], which prompts phospholipase C activation, fostering inositol triphosphate (IP3) formation and elevating cytosolic Ca2+ levels and cAMP production [159,160]. Additionally, melatonin activates Protein Kinase A in *P. chabaudi* [161]. Melatonin regulates 38 *P. falciparum* genes, including those in the ubiquitin proteosome system with the help of PfPK7 kinase [162,163]. Another *P. falciparum* kinase- PfelK1 has been reported to be central for melatonin mediated parasite synchronization [164]. It activates the PfNFYB transcription factor and modulates mitochondrial dynamics by affecting FIS1, DYN1, and DYN2 genes [165]. Melatonin also regulates the expression of a *P. falciparum* nuclear protein- PfMORC [166], that plays a key role in regulating the *P. falciparum* cell cycle. Melatonin presence boosts apSig gene (apicoplast subunit gene) and apicoplast transcript expression [167].

Melatonin suppresses mitochondrial-dependent hepatocyte apoptosis and mitigates liver damage caused by free radicals during murine malarial infection [168]. Melatonin treatment improved survival in infected mice, blocking brain edema, preserving the blood-brain barrier, mitigating histological alterations, and preventing motor and cognitive impairments induced by *Plasmodium* infection during ECM (Table 2) [169]. Melatotosil, that blocks melatonin action on *P. falciparum* leads to enhanced parasitemia [164]. Melatonin receptor antagonist, Luzindole, hampers intraerythrocytic parasite growth (Table 2) by disrupting the calcium oscillation and cAMP increase in asexual stages of *P. falciparum* [170]. Several indole derivatives with structure similar to melatonin, specifically triazine-indole and indole alkaloids, display antiplasmodial activity (Table 2), with IC_50_ in the nanomolar range when tested in vitro against *P. falciparum* [171–174]. Among these, spiroindolone KAE609 (Cipargamin) is currently in Phase II clinical trials [174].

## Kidney

Involved in removing waste products and regulating extracellular fluid volume, serum osmolality, and electrolyte balance [175]. Kidney produces hormones including renin, erythropoietin, and 1,25-dihydroxy vitamin D3 [175].

### Renin

Produced as prorenin by juxtaglomerular cells and activated enzymatically, regulates blood pressure, fluid balance, and electrolytes through the RAAS (Figure 3) [55]. It enzymatically converts angiotensinogen to angiotensin I, which is subsequently transformed into angiotensin II to perform RAAS functions [7,55]. Renin secretion is triggered by low blood pressure, low blood volume, reduced sodium, or sympathetic activation [7,55]. Increased renin levels have been linked to acute kidney injury and mortality in children with severe malaria or sickle cell anemia [176].

### 1,25-Dihydroxyvitamin D3 (calcitriol)

Active form of vitamin D that acts as a hormone [177]. It enhances calcium and phosphate absorption in the gut, supports bone mineralization, and works with parathyroid hormone to regulate calcium levels and prevent bone loss [177]. It also enhances renal reabsorption of these minerals, modulates immune responses, and influences cellular growth and apoptosis [177].

Vitamin D3 impacts both innate and adaptive immunity via the vitamin D receptor (VDR) [178]. VDR polymorphisms are linked to gametocyte levels in *P. vivax* infection, and VDR expression is upregulated in murine *P. chabaudi* infection [178]. Serum vitamin D3 levels are notably lower during falciparum malaria infection and well as in ECM [179–181]. Vitamin D regulates the iron hormone hepcidin, and children with lower Vitamin D levels also have increased risk of iron deficiency, which is common during malaria infection [182,183]. In mouse models, Vitamin D3 supplementation reduces ECM by lowering systemic inflammation, cytokines (IFN-γ, TNF), and improving blood-brain barrier function, while enhancing regulatory T cells and IL-10 [184,185].

Calcitriol and its analog, 22-oxacalcitriol (22-OCT), show antiplasmodial activity against *P. falciparum* in vitro, and during murine *P. chabaudi infection* (Table 2), with in vivo effects that are INF-γ independent [186]. Despite inducing antimicrobials like NO and cathelicidin, the inhibition of *Plasmodium* growth was independent of these factors [187]. Combining arteether with Vitamin D3 improves outcomes in ECM (Table 2) [188]. Additionally, Vitamin D enhances CTL-mediated pathogen defense, reflecting a potential link between Vitamin D and CTL immunity in malaria [189].

### Erythropoietin (Epo)

**Figure 4:**
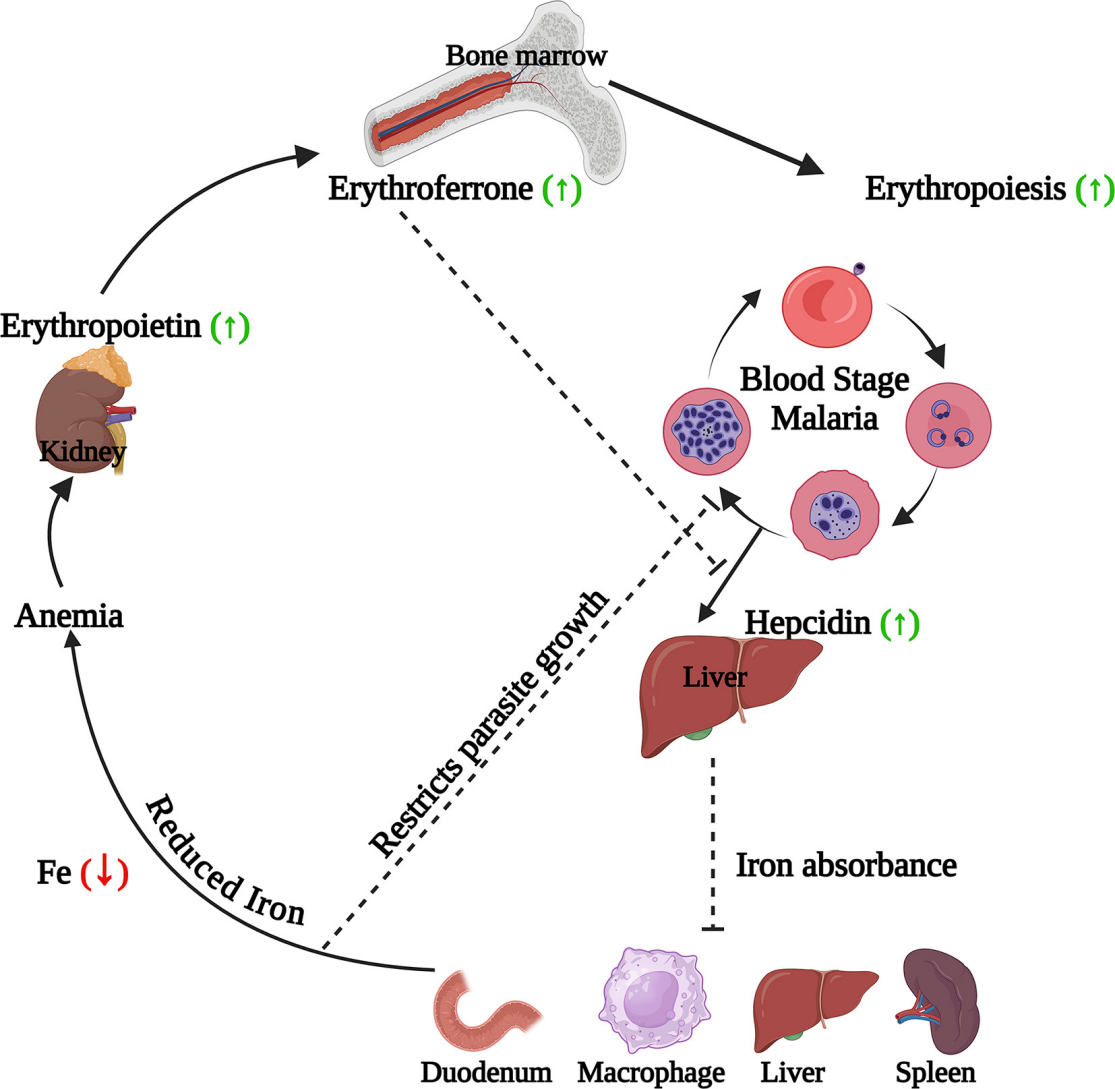
Iron regulation and erythropoiesis during malaria During malaria, *Plasmodium* infection prompts the liver to increase hepcidin production, reducing iron absorption. Although lower iron levels inhibit parasite growth, they also cause anemia. In response, the kidneys release erythropoietin, stimulating erythropoiesis in the bone marrow and activating erythroblasts to produce erythroferrone. Erythroferrone, in turn, inhibits hepcidin to counteract anemia.

Erythropoietin is a kidney-produced hormone that stimulates erythrocyte production (erythropoiesis) in response to anemia or hypoxia (Figure 4) [190]. Erythropoietin levels are generally elevated in individuals with malaria-induced anemia (Figures 1 and 4) [191–196] and cerebral malaria [197]. Higher Epo levels are observed in children with cerebral malaria who have retinopathy compared to those without [198]. However, some studies report low Epo levels in anemic malaria patients with *P. falciparum* [199,200]. Reduced erythropoietin levels have been correlated with fetal anemia [201]. High anti-Epo antibody levels in pregnant women with *P. falciparum* malaria, likely due to parasite density, may contribute to malarial anemia [202]. Similar results are seen in murine malaria, where anti-Epo antibodies correlate with hemoglobin loss [203].

Elevated Epo levels have been observed in other murine malaria studies [204]. In *P. chabaudi* infected C57BL/6 and A/J mice (non-severe malaria), kidney Epo production correlates with anemia severity, regulated by hematocrit levels [205–207]. However, in murine anemia during severe malaria (*P. berghei* infected BALB/c), erythropoietin does not influence anemia onset [208]. Acute anemia in *P. berghei*-infected mice results from increased myeloid cell production and cytokine release, impairing erythroid development, similar to chronic disease-related anemia [191]. Additionally, parasite factors like hemozoin may inhibit Epo-induced erythroid precursor proliferation, contributing to severe malarial anemia [209–211].

Epo treatment alleviates cerebral malaria symptoms and reduces cerebral pathology during ECM [212,213]. However, if given early, recombinant Epo can worsen infections by accelerating reticulocytosis and parasite multiplication [214]. Epo decreases hypoxia and inflammation, has neuroprotective effects, and can reduce mortality in murine models of ECM [215–218]. It suppresses inflammation by inhibiting splenic dendritic cells and promoting regulatory T cells, while also upregulating CTLA-4 [216]. Epo activates neural stem cell progression [215], reduces neuronal apoptosis [219], thus mediating neuroprotection during murine cerebral malaria pathology [190]. Epo treatment in mice also normalizes elevated cerebral and systemic VEGF, along with HIF-1α levels in the brain, that are normally elevated during cerebral malaria [220]. Caspase and calpain (elevated during eryptosis) activity also get significantly reduced in mice treated with Epo [220]. Epo also regulates the expression of hepcidin (Figure 4) (hormone with antimalarial effect) in the livers of *P. berghei* infected mice [221].

High Epo levels are linked to a lower risk of neurological sequelae in children with cerebral malaria and may be considered for adjunct therapy [222]. Moreover, artesunate-erythropoietin treatment has been shown to induce early recovery from *P. berghei* induced malaria as compared to artesunate mono-treatment (Table 2) [223]. Also, Epo doses combined with quinine has been reported safe in short-term malaria treatment (Table 2), with speculation to be tested further in clinical trials [224]. Chloroquine reduces Epo levels in healthy individuals but increases them in malaria patients, likely due to its anti-inflammatory effects [225].

## Liver

The liver is crucial for metabolism and detoxifying xenobiotics [226]. It also has endocrine function, producing hormones such as hepcidin, thrombopoietin, angiotensinogen, and insulin-like growth factors 1 and 2 [226].

### Hepcidin

Hepcidin is a peptide hormone crucial for iron regulation [227]. It decreases iron absorption from the gut and limits iron release from storage sites (macrophages, hepatocytes, and enterocytes) (Figure 4) by interacting with and degrading ferroportin [227]. Hepcidin levels rise with high iron to reduce absorption and release, and fall with low iron to increase availability [227]. Inflammation can also elevate hepcidin, potentially leading to iron deficiency anemia by restricting iron needed for red blood cell production [227,228].

Malaria causes iron deficiency [229,230], with elevated hepcidin levels observed during infection and in children exposed to placental malaria during birth (Figure 4) [231]. Hepcidin levels also correlate with parasite density, IL-10 and IL-6 in acute *P. falciparum* malaria [232,233]. High IL-10 and IL-6 during pediatric malaria boosts hepcidin production [232,233]. A positive association of hepcidin and IFN-γ been reported in severe malaria [234].

In malaria, anemia results from increased erythrophagocytosis and dyserythropoiesis, with elevated hepcidin playing a key role (Figure 4) [228,235]. Elevated hepcidin reduces iron absorption and availability, worsening anemia and causing functional iron deficiency (Figure 4) [228]. However, this also limits iron for *Plasmodium* growth, potentially reducing parasite levels and preventing new infections by lowering liver iron and parasite growth (Figure 4) [236]. This explains why iron deficiency can protect against severe malaria [237] and iron supplementation might increase infection risk. Exogeneous administration of synthetic hepcidin protects against *P. berghei* induced murine malaria (Table 2), as well as exerts anti-inflammatory activities [238].

### Thrombopoietin

A 335 kDa protein that aids megakaryocyte maturation and platelet production, and also supports hematopoietic stem cells and progenitor cells [239]. Platelets has been reported to kill intraerythrocytic malarial parasites [240], and thus during malarial infection thrombocytopenia is very common [241]. In response to thrombocytopenia, elevated thrombopoietin levels has been reported in patients with severe *P. falciparum* malaria [239].

### Angiotensinogen/Angiotensin-I/Angiotensin-II

Angiotensin and the RAAS (Figure 3) regulate blood pressure, fluid balance, and vascular resistance [55,242]. Angiotensin, a peptide hormone, exists as angiotensin I, II, and III [55,152]. Renin converts liver-produced angiotensinogen to angiotensin I, which angiotensin-converting enzyme (ACE) in the lungs converts to angiotensin II (Figure 3) [242]. The active angiotensin II raises blood pressure and increases sodium and water retention (Figure 3) [55].

Malaria infection often leads to changes in blood pressure [2,3], and angiotensin plays a key role in blood-pressure regulation [55]. A hypothesis suggests that genetic polymorphisms in the RAAS (e.g., ACE I/D, ACE2 rs2106809), that got selected for malaria protection, raise blood pressure, linking malaria exposure to increased hypertension risk [243,244]. The Angiotensin II/AT1 (Angiotensin II type 1 receptor) pathway has been reported to enhance renal pro-inflammatory cytokines during ECM, leading to glomerular and tubular injuries seen in Malaria induced acute kidney injury [242]. Angiotensin II also promotes effective splenic T cell activity during *P. berghei* infection [245].

ACE inhibitor- Captopril, elevates mortality of *P. chabaudi* infected mice [246]. In contrast, AT-1 receptor blockers like irbesartan and losartan improve survival in mice with cerebral malaria and enhance the effectiveness of existing antimalarial drugs like arteether (Table 2) [247,248]. Angiotensin II derivatives, such as VIPF and Ang II-SS, protect mice against severe malaria without vasoconstriction (Table 2) [249]. Ang II peptides reduce mosquito salivary gland infectivity and sporozoite infectivity in avian malaria [250,251]. Angiotensin II and the derived compounds can impair *P. falciparum* growth in vitro [252]. This indicates potential antimalarial targets within the RAAS system [247–252].

### Insulin-like Growth Factor 1 (IGF-1)/somatomedin C

IGF-1 is a hormone similar to insulin, mainly produced in the liver in response to pituitary growth hormone, promotes growth and development [7]. IGF-1 plays a key role in immune responses, especially in TH_2_ immunity, and helps maintain blood-brain barrier integrity [253]. It suppresses inflammatory mediators like TNF-α in the central nervous system [254], thereby administration of IGF-1 reduces cerebral malaria induced mortality in mice [255]. Mice with non-cerebral malaria, express high level of IGF-1 [254]. *P. falciparum* infection decreases serum IGF-1 [256]. Placental *P. falciparum* infection reduces IGF-1 in both maternal and neonatal plasma [257]. The cord blood IGF-1 also is found to be lower during maternal malaria [258]. Lower IGF-1 reported in serum of young children with anemia induced during *P. falciparum* infection [259], and speculated to be a reason for reduced hematopoiesis during malaria, thus leading to anemia [259]. Additionally, lower IGF-1 is seen in children with systemic inflammation [260]. Hemozoin due to *Plasmodium* infection, has been reported to inhibit IGF-1 signaling through the MAPK and PI3-K pathways [261]. Additionally, in mosquitoes, IGF-1 in the midgut (ingested during blood-meal) suppresses MAPK ERK phosphorylation, boosting resistance to *P. falciparum* by enhancing reactive nitrogen and oxygen species production [262,263].

## Bone Marrow

### Erythroferrone

Hormone produced by erythroblasts in response to anemia, regulates iron metabolism by inhibiting hepcidin and boosting iron availability for red blood cell production (Figure 4) [264]. During malaria, hepcidin levels are high, but severe anemia in murine models leads to increased erythroferrone and decreased hepcidin levels during later stages of infection [264]. Elevated erythroferrone is also seen in malaria patients with severe anemia [265].

## Intestine

### Glucagon-like peptide-1 (GLP-1)

GLP-1 is an intestinal hormone that aids in glucose metabolism and appetite control [6,7]. It enhances insulin secretion, inhibits glucagon release, slows gastric emptying, reduces appetite, improves insulin sensitivity, and may benefit cardiovascular health [6,7]. GLP-1’s neuroprotective effects involve reducing neuronal death, inflammation, and oxidative stress while enhancing synaptic plasticity and mitochondrial function [266]. GLP-1 receptor agonists are used in diabetes treatment. However, GLP-1 analogue liraglutide failed to protect mice from ECM nor was able to inhibit *P. falciparum* growth in vitro [266].

### Serotonin

Tryptophan is metabolized through two main pathways: the kynurenine pathway, producing kynurenine and its derivatives, and the methoxyindole pathway, converting tryptophan to serotonin and melatonin [267]. Serotonin- a neurotransmitter, regulates mood, appetite, sleep, and behavior [268]. Produced mainly in the brain and gut, its deficiency can lead to mental disorders [267,268]. Tryptophan derivatives, like melatonin, serotonin, N-acetylserotonin N(1)-acetyl-N(2)-formyl-5-methoxykynuramine and tryptamine, synchronize the development of intraerythrocytic malaria parasites [269]. They cross red blood cell membranes, mobilizing intracellular Ca^2+^ to raise cytosolic Ca2^+^ in *P. falciparum* trophozoites, increasing multinucleated forms [160,270,271].

Lower levels of serotonin are found in human brain during malaria infection [71]. Decreased uptake of tryptophan has been reported in cerebral malaria patients, indicative reason for lower levels of serotonin in malaria patients [71,272]. Mice with severe malaria have lower serotonin levels in their serum [181]. *P. berghei* -infected mice show decreased serotonin in liver, lung, spleen, and brain but increased levels in kidney and intestine [273]. C57BL/6 mice, more susceptible to *P. berghei* than Swiss albino mice, have lower brain levels of dopamine, epinephrine, norepinephrine, and serotonin during malaria [274]. Increased serotonin helps lower body temperature and aids in cerebral vasodilation during malaria [275,276].

*Two of the* meridianin and psammopemmin analogs (4-Methoxymeridianin A and 20 -debromo-20 -chloro analog of psammopemmin) that could bind with the serotonin receptors, could inhibit *P. falciparum* growth in vitro (Table 2) [277]. A serotonin receptor antagonist-dihydroergotamine methanesulfonate has also been reported to inhibit *P. falciparum* growth in vitro (Table 2) [278]. In another study, three of serotonin receptor agonists: 2,5-dimethoxy-4-iodoamphetamine, 2,5-dimethoxy-4-bromophenylethylamine, and 8-hydroxy-N-(di-n-propyl)-aminotetralin (8-OH-DPAT) reduced *P. falciparum* growth in vitro, with strain transcending growth inhibitory activity seen for 8-OH-DPAT (Table 2) [279]. 6-bromoaplysinopsin, identified to be a ligand of serotonin receptor 5-HT2, also exhibits antiplasmodial activity in vitro (Table 2) [280]. Another molecule with anxiolytic properties: TCMDC-139046, which interacts with serotonin antagonist receptors 5-HT2, has been reported with antimalarial efficacy when tested against *P. falciparum* in vitro (Table 2) (IC50 in the nM range) [281]. Citalopram a particular 5-HT reuptake inhibitor has been reported to reverse the chloroquine resistance of *P. falciparum* and *P. chaubadi* (Table 2), significantly decreasing the IC50 [282,283]. p-chlorophenylalanine (serotonin synthesis inhibitor) and cyproheptadine (serotonin, bradykinin and histamine antagonist) has been reported to reduce the parasitemia load when assessed either separately or with chloroquine for *P. yoelii nigeriensis*-induced malaria in Swiss albino mice (Table 2) [284]. A specific SERT (serotonin uptake transporter present on platelets and neurons) inhibitor-Prozac, has been reported to delay the start of ECM, though did not stop the neuropathology [285]. However, fluoxetine- another 5-HT reuptake inhibitor antagonizes the antimalarial efficacy of primaquine when tested on *P. berghei* infected C57BL/6 mice, even though fluoxetine inhibits the metabolism of primaquine by the enzyme-CYP 2D6 [286].

Quinine decreases serotonin production by inhibiting tryptophan hydroxylase [287,288]. Mefloquine has been reported to act as a partial 5-HT2A (serotonin receptor) agonist and a full 5-HT2C (serotonin receptor) agonist [289]. The antimalarial drugs- Quinine, chloroquine and mefloquine; act as antagonists to 5-HT3 receptors, thus antagonizing the serotonin (5-HT) mediated contractions of the gastro-intestinal tract [290].

When *A. stephensi* mosquitoes are fed blood with low serotonin, their flight speed, object investigation, blood-feeding tendency, and infection success with *P. yoelii-*17XNL increase [291]. Treatments with fluoxetine, high serotonin, and methiothepin decrease these behaviors, indicating the serotonergic networks can be a target for vector control [292]. Serotonin and glutamate have also been reported to enhance the heart contraction rate of *A. gambiae* mosquitoes [293].

## White adipose tissue/adipocytes

### Leptin

Hormone released by adipocytes in response to nutrition, signals the CNS and peripheral organs [294]. Circulating leptin levels are influenced by body fat, metabolic hormones, and gender, with women typically having higher levels [294]. It regulates metabolism, affecting glucocorticoids, insulin, and energy balance [294]. Leptin plays a crucial role in immunity, with leptin’s deficiency can increase susceptibility to infections [294].

In *P. berghei*-infected mice, serum and urine leptin and soluble leptin receptor levels increase [295]. Conversely, malaria patients show decreased blood leptin, with leptin being suggested to be used as one of the prognostic markers of malaria [296–299]. Leptin exacerbates ECM pathology, with a leptin-antagonist peptide that antagonizes leptin signaling, protects against ECM and enhances mice survival [300]. mTORC1, a downstream target of leptin in T cells, when inhibited, can shield against ECM by altering T cell function [300]. In ECM, infected erythrocyte sequestration in white adipose tissue increases local vascular permeability and leptin production [301]. In human subcutaneous adipose tissue, parasite sequestration, phosphorylation of mTORC1, and local leptin production correlate with cerebral malaria mortality, despite unchanged circulating leptin levels [301].

### Adiponectin

Adiponectin reduces insulin resistance and has anti-atherogenic, anti-inflammatory, and anti-diabetic effects [302]. Cerebral malaria patients have high plasma adiponectin levels compared to uncomplicated malaria [139]. Additionally, plasma adiponectin was positively correlated with glucose production and gluconeogenesis in these patients [139].

## Conclusion

Malarial parasite infection, disturbs the physiological homeostasis of the host, which in turn is maintained by the neuroendocrine system [2–4]. Malaria infection dysregulates the hypothalamus-pituitary-adrenal [6,25,27–36], hypothalamus-pituitary-gonad [6,94,111,114] and hypothalamus-pituitary-thyroid axis [6,23,73,75,76] (Figure 1). Malaria seems to stimulate the HPA-axis that leads to over-production of hormones like cortisol, aldosterone and epinephrine [16,28–39,56,57] (Figure 2). Cortisol, being immunosuppressive reduces inflammation but promotes parasite survival [20,25,30,40], whereas epinephrine can mitigate the hypoglycemic effect of malaria [69,70] (Figure 2). During malaria infection, HPG and HPT axis also gets depressed, with reduced levels of sex hormones (estrogen, progesterone and testosterone) and thyroid hormones (T4) being reported [6,23,73,75,76,93,111,114] (Figure 2). The RAAS pathway gets affected during malaria, with enhanced angiotensin-II and aldosterone level being observed, that explains for high edema and hypertension in malaria patients [55,57,58] (Figure 3).

Malaria infection leads to iron deficiency and anemia [2,3,229,230]. Enhanced hepcidin levels observed in patients reduces iron absorption, thus reducing iron availability required for parasite growth [227,228,236,237] (Figure 4). However reduced iron availability will lead to lesser erythrocyte formation, leading to anemia [227–231,235]. In response to malaria induced anemia, erythropoietin levels are found to be higher in malaria patients, which in turn promotes erythrocyte formation and stimulates erythroferrone (negative regulator of hepcidin) production [191–198,264,265] (Figure 4). However, other parasite factor like hemozoin may inhibit Epo-induced erythroid precursor proliferation [209–211].

Hypoglycemia is more common in malaria, that is attributed to reduced glucose production and hyperinsulinemia [136–138]. However, in certain cases hyperglycemia has also been observed, where malaria induced stress hormones and pro-inflammatory cytokines can lead to lower insulin production or insulin resistance [139–144]. Hypocalcemia and hypophosphatemia are common during malarial infections, that can be attributed to low Vitamin-D3 and parathyroid hormone, but higher calcitonin in malaria patients [5,78–83,90,179–181].

Since, hormones can directly or indirectly modulate the growth of malaria parasite, few of them have been looked upon as probable antimalarials or targets for antimalarial therapies. Exogeneous administration of hormones like DHEA, melatonin, PTH, Vitamin-D3, hepcidin, progesterone and erythropoietin have been reported to protect against malaria pathologies [63–66,93,168,169,184,185,212,213,215–218,238]. Thereby, the synthetic analogs, mimics, and receptor agonists or antagonists of some of these hormones are been evaluated to be used either as antimalarial or as adjunct therapy against malaria [67,112,170–175,186,188,223,224,277–284]. Angiotensin-II peptide mimics too have been reported with antimalarial activity [249–252]. Additionally, somatostatin analogs help prevent hyperinsulinemic hypoglycemia during malaria complications [11]. Moreover, hormones influence the immune system and thus can regulate the severity of infection [20,25,30,40,41,59,60,99–102,117–119,184,185,216,254], with leptin antagonists and cortisol modulators been explored to target hormonal pathways against malaria [46–52,300]. It is important to note that most studies on parasite growth inhibition have been conducted either in vitro using the human malaria parasite *P. falciparum* or in rodent models of malaria infection that do not involve human malaria parasites [46–52,63–67,93,112,168–175,184–186,212,213,215–218,223,224,238,277–284,300]. Therefore, it remains to be seen how many of these findings will progress through clinical trials to substantiate the hypothesis that hormones or their analogs could be used as antimalarials.

Moreover, few hormones like PCT and leptin, have also been explored as markers of malaria infection [81–83,296–299]. Since host hormonal imbalances are common during most infections, identifying unique hormone parameters for malaria as diagnostic markers would be a challenge and may require the inclusion of other molecules that may enhance the diagnostic specificity [81–85,296–299].

To summarize, research done over the last few decades on understanding the endocrine-interplay during malaria infection, has led us to a greater understanding of the intricate relationship between hormones and malaria, that provides valuable insights into disease mechanisms and highlights potential therapeutic targets or molecules [4,9–23,25–54,56–58,61–76,78–126,129–151,153–174,178–225,227–231,233–260,262–266,268–302]. Research has predominantly concentrated on a limited number of hormones, leaving significant areas [4,9–23,25–54,56–58,61–76,78–126,129–151,153–174,178–225,227–231,233–260,262–266,268–302] unexplored. Future research to explore hormonal influences on malaria may lead to targeted interventions that can mitigate severe disease and enhance patient outcomes.

## Abbreviations

CRH: corticotropin-releasing hormone
ECM: experimental cerebral malaria
GHRH: growth hormone-releasing hormone
GnRH: gonadotropin-releasing hormone
HPA: hypothalamic-pituitary-adrenal
HPG: hypothalamic-pituitary-gonadal
HPT: hypothalamic-pituitary-thyroid
TRH: thyrotropin releasing hormone
TSH: thyroid stimulating hormone
